# Strategic Purchasing: The Neglected Health Financing Function for Pursuing Universal Health Coverage in Low- and Middle-Income Countries: Comment on "What’s Needed to Develop Strategic Purchasing in Healthcare? Policy Lessons from a Realist Review"

**DOI:** 10.15171/ijhpm.2019.34

**Published:** 2019-05-29

**Authors:** Kara Hanson, Edwine Barasa, Ayako Honda, Warisa Panichkriangkrai, Walaiporn Patcharanarumol

**Affiliations:** ^1^ Faculty of Public Health and Policy, London School of Hygiene and Tropical Medicine, London, UK.; ^2^ KEMRI-Wellcome Programme, Nairobi, Kenya.; ^3^ Department of Economics, Sophia University, Tokyo, Japan.; ^4^ International Health Policy Program, Ministry of Public Health, Nonthaburi, Thailand.

**Keywords:** Strategic Purchasing, Universal Health Coverage, LMICs, Health Financing, Provider Payment, Benefit Package

## Abstract

Sanderson et al’s realist review of strategic purchasing identifies insights from two strands of theory: the economics of organisation and inter-organisational relationships. Our findings from a programme of research conducted by the RESYST (Resilient and Responsive Health Systems) consortium in seven countries echo these results, and add to them the crucial area of organisational capacity to implement complex reforms. We identify key areas for policy development. These are the need for: (1) a policy design with clearly delineated responsibilities; (2) a task network of organisations to engage in the broad set of functions needed; (3) more effective means of engaging with populations; (4) a range of technical and management capacities; and (5) an awareness of the multiple agency relationships that are created by the broader financing environment and the provider incentives generated by multiple financing flows.

The academic literature on strategic purchasing, together with a related literature on contracting of health services, has long adopted principal-agent and transaction cost economics as its main theoretical underpinning.^1^ The recent paper by Sanderson et al^2^ substantially augments the traditional theoretical perspectives on strategic purchasing by adding insights from the literatures on the theories of inter-organizational relationships. They argue that the economics of organisation brings awareness into the “safeguarding” element of strategic purchasers – how best to design contractual and extra-contractual arrangements to structure interactions among patients, purchasers, government and providers, in order to create incentives to provide responsive, equitable and efficient care. However, the inter-organizational relationships literature helps to understand the challenges of coordination and adaptation within strategic purchasing relationships, recognizing the need for cross-organizational working to achieve value in the health system, and also that trust and collaboration are needed. Having a strong theoretical grounding is essential, they argue, for transfer of policy ideas from one setting to another to be informed by a clear understanding of both the mechanism that is influencing behaviour, and the context within which policies are implemented. In a world where decisionmakers at the global and national levels are increasingly looking to learn from other countries’ experience, the realist evaluation approach, which emphasises the importance of context – mechanism – outcome to understand “what works where, for whom” is a potentially powerful approach. However, as Sanderson et al note, nearly all of the empirical work on which they draw to illustrate their theoretical insights is from high income settings.

From its beginnings in the United Kingdom and European literature,^1^ the term strategic purchasing is now firmly lodged within the global health policy lexicon: it has been the subject of international meetings^3^ and of major programme investment by some donors, for example the Strategic Purchasing Africa Resource Centre.^4^ It also increasingly features in national policies in low- and middle-income countries (LMICs), particularly as governments set out to develop their strategies to achieve Sustainable Development Goal (SDG) 3.8, universal health coverage, providing equitable access to quality health services without risk of financial hardship. Strategic purchasing is mentioned in national policy documents and plans in places such as Kenya, South Africa, Ghana, and Nigeria. This has been a critical policy development because up to now, health financing policies have primarily focused on resource generation and not on how resources are transferred to providers. Failure to focus on the key purchasing questions – what services should be covered, which providers should deliver them, and how should they be paid for – presents a risk that any increased coverage or spending will fail to provide the reductions in out-of-pocket payments and improved financial protection that universal health coverage promises. Further, when purchasing arrangements are not intentional about enhancing desired health system goals, the quality, efficiency, and equity of health systems may be compromised. Strategic purchasing provides the critical link between health financing policies and health service delivery. But the design and successful implementation of this complex policy is a challenge.

RESYST (Resilient and Responsive Health Systems) was a research programme consortium funded by the UK Department for International Development (https://resyst.lshtm.ac.uk). From 2010 to 2018, RESYST members conducted policy-oriented health systems research on three themes – health financing, health workforce, and governance and leadership. Strategic purchasing was a key topic area of research within the consortium. Like many others working in the field, we also adopted an initial analytic framework that drew from principal agent theory, seeking to explore the way that information, incentives, decision-making authority and accountability shaped the relationships between a purchaser and the three other key actors they work with – government, providers, and the population. Altogether we studied 19 purchasing mechanisms in 10 countries. These included integrated purchaser-provider relationships in tax funded systems (for example, the tax funded system in South Africa, Nigeria and Tamil Nadu, India), “public contract” models in which public funds (either tax or social health insurance) were transferred between purchaser and providers in a more arms-length relationship (such as the Universal Coverage Scheme in Thailand, Vietnam Social Security, and the BPJS scheme in Indonesia), and “private contract” models in which private health insurers purchased services from private providers (such as private medical schemes in South Africa and private insurers in Kenya).^5^ For each purchasing mechanism we sought to critically assess its performance and identify the factors influencing it.

In setting out to describe and assess the effectiveness of the purchasing relationship in these different settings, a first barrier that we encountered was a lack of clarity as to what activities were involved in strategic purchasing. To fill this gap we developed a short policy note^6^ that elaborated the different actions that a strategic purchaser would take in relation to government, providers and citizens. Mindful that there is often a gap between policies that exist on paper and actual implementation, we reviewed written policies and other key documents, and conducted key informant interviews and focus group discussions to identify where policies existed and did not, in relation to these strategic purchasing actions; ascertained the extent which these actions were actually taking place; and identified factors influencing the implementation gaps.

From looking across this large set of purchasing relationships, we found that much purchasing was not strategic, but rather, that it remained passive. The formulation of the benefit package that was being purchased often failed to take into account both cost-effectiveness and citizen preferences, did not employ an evidence informed and transparent process, and the package itself was loosely described, providing little opportunity to obtain value from health spending. The selection of providers was often passive: in the case of public providers it was difficult, if not impossible, to exclude them from the purchasing system even if the quality or value for money they offered was low, because of lack of alternative providers in rural and remote areas, and because the public financial management frameworks did not allow public facilities to be excluded from funding arrangements. For instance, the Vietnam Social Security fund was obliged to provide contracts to all public healthcare facilities based on a list approved by the Ministry of Health every year.^7^ Contracting arrangements were generally passive, with little adoption of more high powered payment mechanisms and little use of the purchaser’s power to set quality standards and enforce them. Finally, purchasers in most countries had weak systems to engage citizens and to be accountable to them. For instance, Kenya’s National Hospital Insurance Fund (NHIF) had a complaints and feedback telephone number that was not functional in practice making it impossible for citizens to place complaints and feedback to the NHIF.^8^ There were some clear exceptions to this – the Universal Coverage scheme in Thailand being a notable example^9^; and many countries were beginning to identify ways to incrementally make their purchasing arrangements more strategic, for example by introducing health technology assessment to inform benefit package choices, and planning provider payment reforms such as the introduction of capitation in Kenya, Vietnam, and Nigeria.

By providing rich empirical evidence from a wide range of LMIC settings, a number of these findings can be framed by the theoretical insights of the Sanderson et al review.

First, the agency theory perspective points to the need for a clear policy design, to enable the definition and communication of the roles and responsibilities of healthcare purchasing actors and shape the mindset of an organisation and its staff, so that reforms can be implemented smoothly. For example, in Indonesia, when the Social Health Insurance scheme was introduced in 2014, the roles of the various actors in the system were unclear and there was confusion among the central-level public purchaser, the Ministry of Health, the District Health Office and local government about who would audit and supervise the central level purchaser, who would pay primary healthcare providers, who would monitor healthcare providers, and to whom public providers were accountable.^10^

Second, mature strategic purchasing systems drew on a broad “task network”^11^ of agencies and bodies who can take responsibility for activities supporting or strengthening purchasing functions. In order to be effective, this expanded set organisations need to be carefully coordinated (a relational issue) and guided by a clear policy framework (see above). In Thailand, for example, health technology assessment to inform modification to the benefit package is undertaken by the Health Intervention and Technology Assessment Programme, the International Health Policy Programme, both semi-autonomous research agencies under the Ministry of Public Health, and other Thai universities. The Healthcare Accreditation Institute accredits public and private healthcare providers that are contracted to provide services under the Universal Coverage Scheme. Departments at the Ministry of Public Health take responsibility for improving service quality. The National Health Commission Office supports civil society involvement in healthy public policies through the mechanism of a health assembly and an annual public hearing for National Health Security Office (NHSO). The Thai Health Promotion Foundation (ThaiHealth), financed by a 2% surcharge on excise tax levied on tobacco and alcohol, manages the Health Promotion Fund which supports all relevant sectors, public, private and civil society, to carry out active health-promoting activities. These additional organisations make the environment more complex in terms of managing the network relationships, but they also bring in a broader range of capacities (see below).

Third, a common failing of purchasing systems was ineffective engagement with citizens and patients. Sanderson et al point to theoretical insights from inter-organisational relationships theory about trust and interaction as necessary underpinnings for such engagement to make healthcare systems more responsive to patient needs and preferences. This involves both eliciting population health priorities (needs and wants) and finding ways to effectively channel patient power (through voice or choice) to encourage good performance by providers. In most of our study countries, the systems for consulting with populations were *ad hoc* and irregular, and there was no clear mechanism to channel the information gathered into a process of benefit package development. But population awareness of the systems for gathering patient feedback was also limited, and the passive mechanisms adopted, such as patient feedback boxes, were mostly unused. Thailand was again an exception, with both robust systems for involving patient interest groups (albeit often focused around particular diseases) but also a well-functioning telephone helpline, aimed at providing guidance to scheme members on how to access their entitlements and also functioning as a feedback mechanism to receive patient complaints.

One empirical finding which is not directly anticipated in either the economics of organisation or the inter-organisational relationships literatures was the critical importance of organisational capacity. Strategic purchasing is technically demanding and requires a number of capacities of the purchaser. We examined the development of the NHSO in Thailand, recognised as an exemplary strategic purchasing organisation that was created to administer the Universal Coverage Scheme in 2002. Our analysis identified the core technical capacities that have enabled the NHSO to manage the purchasing function in such a way as to increase access to services, provide financial protection, monitor the quality of care, assure value for money, protect consumer rights and to do this in a sustainable manner. These technical capacities are shown in the Figure. Organisational capacity is often neglected in the design and implementation of complex healthcare reforms, yet has an important influence on their success. Greater attention to both aligning reforms with capacity, and to developing the capacity of implementing organisations to execute these new functions, will be essential for the success of purchasing reforms.

**Figure:**
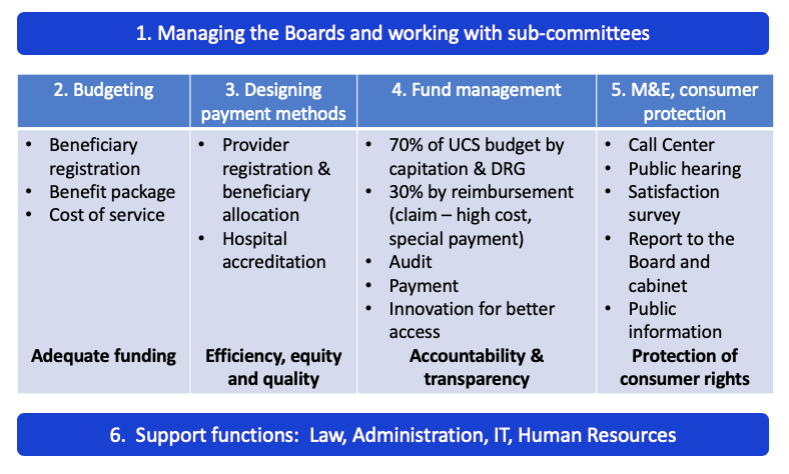
Capacities Required for a Strategic Purchaser. Abbreviations: DRG, diagnosis-related group; M&E, monitoring and evaluation.
(Source: International Health Policy Programme presentation to RESYST annual meeting, October 5, 2018).

A second area where our work extends the findings of Sanderson et al is in relation to the agency relationships between purchaser and providers. Most studies of strategic purchasing have taken the perspective of the purchaser and a single flow of funds; this is exemplified in our own studies which describe the benefit package, payment mechanism and contracting arrangements for each individual funding flow. However, in practice health financing systems are characterised by multiple funding flows, each of which is attached to a specific service package, provider payment mechanism, and set of reporting requirements. This creates a series of parallel, multiple agency relationships that must be navigated by the provider. For a healthcare provider operating in the context of the fragmented funding environment that is typical of LMICs, this means that they may face a number of distinct funding flows, each sending different signals about what activities they should focus on, and different incentives to serve specific population groups or to use resources in a certain way. For example, a typical healthcare provider in Kenya would receive some funding in the form of a line item budget from the county government, capitation payments from the NHIF for civil servants, capitation payments from the NHIF at a different payment rate for the national scheme, case based payments from the NHIF for the Free Maternity programme for poor women, user fees from patients, and potentially drugs or supplies in kind for vertical programmes supported by donors. In this noisy financing environment, it is hardly surprising that providers have difficulties prioritising and using their resources efficiently. But such arrangements are also potentially highly inequitable, leading to differential quality of care for different groups, depending who is paying for them. This complex agency environment can also facilitate opportunistic behaviour by providers.

Realist review, as conducted by Sanderson et al, is a promising approach to integrate theoretical insights with empirical observation in order to inform policy transfer. By broadening the theoretical framings of strategic purchasing to include inter-organisational relations, their paper adds new insights into the factors that are likely to influence the effectiveness of this complex policy in different settings. Re-interpreting some of our empirical findings, drawn from a variety of settings, along these lines has generated new understanding of some of the challenges that our study countries were facing. Theories of government capacity would provide additional insights which could aid in assessing the transferability of such policies across diverse settings.

## Ethical issues

Not applicable.

## Competing interests

Authors declare that they have no competing interests.

## Authors’ contributions

Conception and design (KH, EB, AH, WP, WP). Analysis and interpretation of data (KH, EB, AH, WP, WP). Drafting of the manuscript (KH). Critical revision of the manuscript for important intellectual content (EB, AH, WP, WP). Obtaining funding (KH).

## Authors’ affiliations

^1^Faculty of Public Health and Policy, London School of Hygiene and Tropical Medicine, London, UK. ^2^KEMRI-Wellcome Programme, Nairobi, Kenya. ^3^Department of Economics, Sophia University, Tokyo, Japan. ^4^International Health Policy Program, Ministry of Public Health, Nonthaburi, Thailand.

## Funding

The authors are members of the Consortium for Resilient and Responsive Health Systems (RESYST). This article is an output from a project funded by the UK Aid from the UK Department for International Development for the benefit of developing countries. However, the views expressed and information contained in the article are not necessarily those of, or endorsed by, Department for International Development, which can accept no responsibility for such views or information or for any reliance placed on them. Funding for the work described in the commentary was also provided by the Asia-Pacific Observatory on Health Policies and Systems.
